# Changes of Peripapillary Retinal Nerve Fiber Layer in Childhood Glaucoma: A Systematic Review and Meta-Analysis

**DOI:** 10.3389/fmed.2021.740152

**Published:** 2021-10-11

**Authors:** Yuwen Wen, Yingting Zhu, Yehong Zhuo

**Affiliations:** State Key Laboratory of Ophthalmology, Zhongshan Ophthalmic Center, Sun Yat-sen University, Guangzhou, China

**Keywords:** childhood glaucoma, retinal nerve fiber layer (RNFL) thickness, glaucoma optic nerve damage, spectral-domain optical coherence tomography (SD-OCT), meta-analysis

## Abstract

**Objectives:** Retinal nerve fiber layer (RNFL) thickness has been detected by numerous studies about alterations and abnormalities in childhood glaucoma, but these studies have yielded inconsistent results about the RNFL thinning region. The investigation of characteristics of RNFL in pediatric patients would contribute to the deep understanding of the neuropathic mechanisms of childhood glaucoma. Thus, the degree of thinning in different quadrants deserves further discussion and exploration.

**Method:** A systematic literature search was conducted using the Cochrane Central Register of Controlled Trials, Medline, Embase, and PubMed databases to identify clinical studies published from inception to April 1, 2021.

**Results:** Ten studies were included in this review with a total of 311 children with glaucoma and 444 in nonglaucomatous controls. The results revealed that average peripapillary RNFL (pRNFL) thickness was attenuated in pediatric patients with glaucoma [weighted mean difference (WMD) = −20.75; 95% CI −27.49 to −14.01; *p* < 0.00001]. Additionally, pRNFL thickness in eight quadrants (superior, inferior, temporal, nasal, superotemporal, inferotemporal, superonasal, and inferonasal) had different levels of reduction in the pediatric group of glaucoma.

**Conclusion:** This study indicates that eight regions of RNFL thickness show various degrees of thinning in childhood glaucoma. However, caution is required in the interpretation of results due to marked heterogeneity. Future studies, especially larger samples and multicenter, need to confirm our results.

## Introduction

Glaucoma is a multifactorial, progressive optic neuropathy with unclear pathogenesis characterized by visual field deficits and cupping of the optic nerve (1–3). As a major cause of irreversible blindness, more than 70 million people worldwide are affected and approximately 10% of the patients with glaucoma are bilaterally blind (4, 5). Childhood glaucoma is a varied group of disorders occurring in children and adolescents younger than 16 years that requires careful attention to prevent vision loss throughout life. It is estimated that 5% of blindness in children worldwide is caused by childhood glaucoma (6). Primary congenital glaucoma (PCG) is the most common type of childhood glaucoma accounting for 32–47% (7, 8) and 70–80% of cases are bilateral (9, 10). Most of the patients with childhood glaucoma present within 6 months of birth with ~ 80% appearing before 1 year of age (11). Childhood glaucoma and its management not only have a marked impact on functional vision of children, but also reduced the quality of life of the patients, relatives, and caregivers (12–14). Hence, early diagnosis is crucial for children affected with glaucoma to receive timely and appropriate treatment.

The clinical visual field is regarded as the essential part of following-up and identifying neurological damage and progressive change in glaucoma. However, visual field testing in children is limited. Different from the adult patients, children have shortened duration for maintaining attention and learning curve, which requires correctly explaining the visual field findings (15, 16). The measurement of intraocular pressure (IOP) is another essential part of the diagnosis and follow-up of glaucoma. However, this detection of young children often has large fluctuation due to poor cooperation.

The pathological changes in glaucoma include cupping of the optic disk, thinning of the retinal nerve fiber layer (RNFL), and loss of the retinal ganglion cells (3, 17, 18). Recently, detection of glaucomatous structural changes has relied on the assessment of morphological changes within fundus photography or direct ophthalmoscopes such as cup-to-disk ratio (CDR) and bilateral asymmetry. A well-known phenomenon in pediatric glaucoma is that cupping of the optic disk can be reversible after remarkable IOP reduction, especially before 1 year of age (19–21). However, cupping reversal in childhood glaucoma may not be a good indicator to assess the improvement of optic nerve head health. Continued RNFL thinning was observed in some cases with IOP reduction and cupping reversal after treatment of glaucoma (22). Preoperative RNFL thickness is a key prognostic indicator of RNFL thinning progress even after surgery and visual prognosis. A precise evaluation of the RNFL is crucial for diagnosing and monitoring pediatric glaucoma.

With the appearance of spectral-domain optical coherence tomography (SD-OCT), it is possible to monitor the automatic segmentation of the individual retinal layers. It has been widely used in the diagnosis and follow-up of glaucoma in recent years. Being a simple, non-invasive, and safe imaging test, it has been intensely applied to measure and observe the change in the peripapillary retinal nerve fiber layer (pRNFL) (23). SD-OCT has dramatically improved image resolution and reliably detects glaucoma disease and progression in adults (24). Considering the difficulty of testing children, the most recent SD-OCT is designed to handheld OCT or overhead mounted OCT to make infants and young children benefit from this technology.

Recently, several studies using SD-OCT were performed to compare pRNFL thickness in children with glaucoma and healthy children and to convey conflicting results. Some researchers have reported the thinning of average pRNFL thickness in all sectors between pediatric glaucoma and controls (25–30). However, other studies have found that children with glaucoma and normal participants have comparable pRNFL thickness in some regions such as the temporal, nasal, and inferior nasal quadrants (31–34). Thus, the possible association of each sector between pRNFL thickness and childhood glaucoma remains unclear, which justifies the need for more studies on this issue. In this meta-analysis, we aimed to carry out a comprehensive overview of the characteristics of thinning RNFL in childhood glaucoma offered by SD-OCT applications. Furthermore, by analyzing the average eight quadrants of pRNFL thickness changes in pediatric glaucoma compared with the normal participants, we can investigate whether pRNFL thickness measurements are affected in childhood glaucoma and whether they could provide a diagnostic tool in assessing pediatric patients as in adults. Finally, this study also summarized similarities and differences of RNFL thickness between children and adults and the role of SD-OCT in childhood glaucoma.

## Materials and Methods

This review was conducted and reported according to the guidelines presented by the PRISMA statements and the Meta-Analyses and Systematic Reviews of Observational Studies statement (35, 36).

### Literature Search

We searched Cochrane Central Register of Controlled Trials, Medline, Embase, and PubMed from database inception to April 1, 2021, with keywords: “childhood glaucoma,” “pediatric glaucoma,” “juvenile glaucoma,” “retinal nerve fiber layer,” “RNFL,” “spectral-domain optical coherence tomography,” and “SD-OCT.” Manual searching of reference lists from all relevant retrieved articles was conducted to identify additional eligible studies. Eligible papers were screened by two authors independently and the duplicated and irrelevant studies were removed. After screening the abstracts, the remaining articles were checked by full-text review.

### Inclusion and Exclusion Criteria

**Figure 1:**
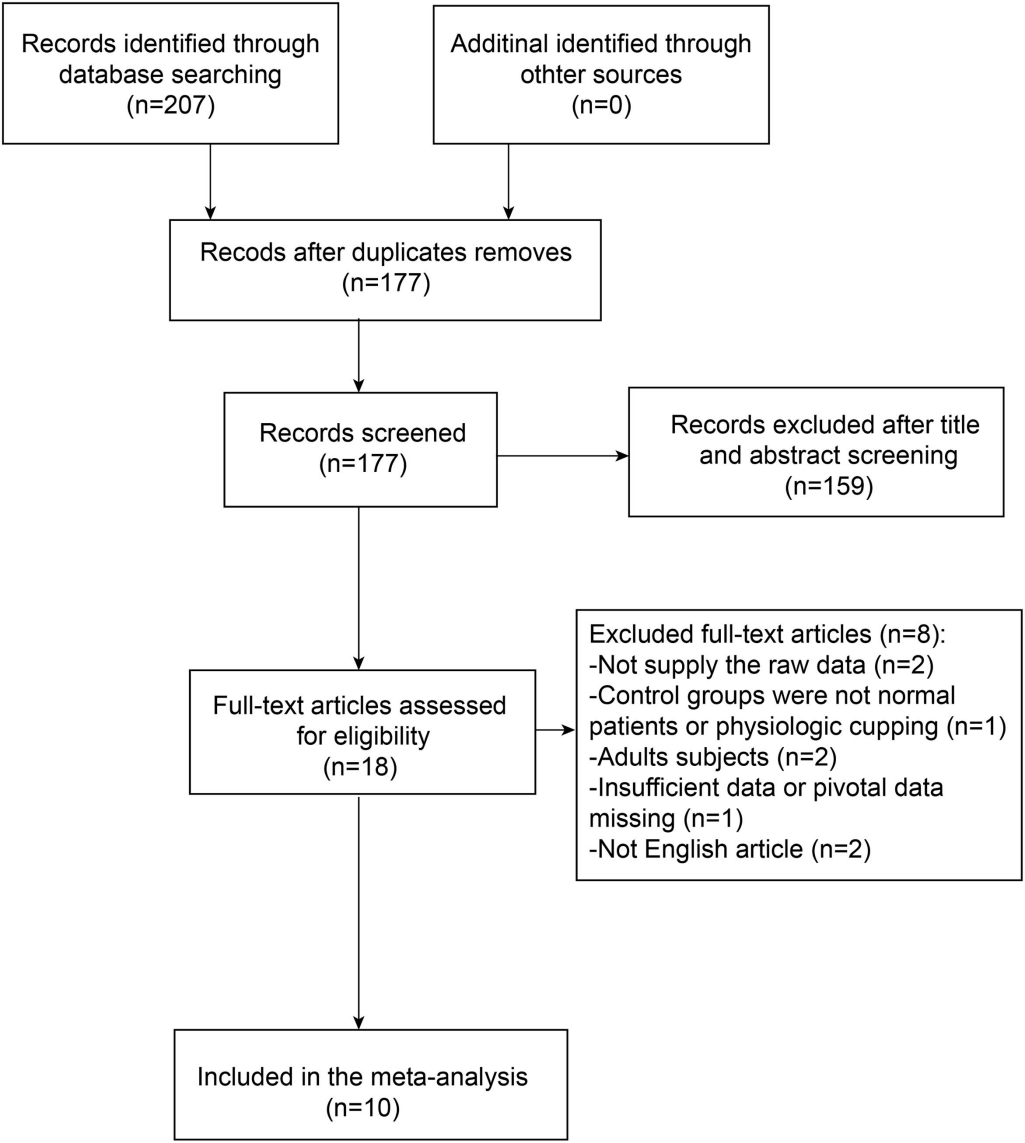
Flowchart of study selection.

We enrolled the studies if all the inclusion criteria and no exclusion criteria were met as follows. The relative search and selection were shown as a flowchart (Figure 1).

In our meta-analysis, the inclusion criteria were (1) original articles reporting independent studies; (2) images with satisfactory quality that measured RNFL thickness by using SD-OCT; (3) satisfactory SD-OCT scan quality recorded in the article; (4) clinical trial, prospective or retrospective cross-sectional study, or case-control study; (5) comparison of pediatric patients with glaucoma and healthy controls or physiologic cupping controls; and (6) sample size ≥10 in each group. Moreover, studies were excluded if they met the following criteria: (1) the study groups were adult patients (aged 16 years or older); (2) RNFL thickness was not measured by SD-OCT; (3) reviews, letters, case reports, and studies with important data unavailable; and (4) studies without the healthy control group.

### Data Extraction

The following information contained in all the eligible publications was retrieved independently by two authors: first author, country, year of publication, study type, SD-OCT manufacturer, glaucoma type, mean age, gender, CDR, number of participations, IOP, and average eight quadrants of pRNFL thickness. Discrepancies in data extraction were solved by discussion or by consulting the third author. Projecting a circle of 3.5 mm in diameter on the retina and divided into eight sectors totaling 360°. The pRNFL thickness parameters evaluated in these studies were average thickness (360° measurement), superior quadrant thickness (46°-135° including 45° superonasal sector and 45° superotemporal sector), inferior quadrant thickness (226–315° including 45° inferonasal sector and 45° inferotemporal sector), nasal quadrant thickness (136–225°), and temporal quadrant thickness (316–45°).

### Quality Assessment

The Newcastle–Ottawa Scale (NOS) was used to assess the methodological quality of all the included studies. The NOS is a star system to judge quality based on three elements of a study: selection, comparability, and outcome or exposure. The maximum NOS score is nine and the study with a NOS score of ≥6 was considered to be of relatively high quality. Quality was assessed independently by the two reviewers and disagreements were resolved *via* discussion.

### Statistical Analysis

We used Review Manager version 5.3 to perform the meta-analyses. The data of RNFL thickness were entered as a continuous variable. Means and standard deviations (SDs) were used to calculate the weighted mean differences (WMDs) for continuous outcomes. A *p* < 0.05 (*p* < 0.05) was regarded as the criterion for statistical significance. Substantial heterogeneity was defined as I2 values of more than 50%. We adopted a fixed effects model for analysis if there was no heterogeneity across studies (*p* > 0.1, I2 <50%). Otherwise, a random effects model was used (DerSimonian and Kacker). The potential publication bias was evaluated by funnel plot and Egger's statistics.

## Results

### Selection of Studies

The literature screening strategy was summarized and presented by a flowchart (Figure 1). In total, 207 pieces of literature were initially identified via the original search and 30 pieces were excluded due to duplication. In addition, 18 publications were left for further evaluation after screening titles and abstracts. Out of these reports, eight pieces could not provide useful or available data for meta-analysis. Thus, we excluded these publications that did not meet the inclusion criteria. Finally, a total of 10 studies, consisting of 311 patients with glaucoma and 444 controls, were included in the meta-analysis.

### Characteristics and Quality Assessment of the Studies

During the enrollment period, a total of 755 participants (311 in the glaucoma group and 444 in the control group) were included in our meta-analysis. Six studies were performed in the USA and the other four studies were performed in the UK, Spain, India, and Germany. The characteristics and qualities of these studies are shown in Table 1.

**Table 1:** Characteristics of included studies.

**Reference**	**Country**	**Study type**	**SD-OCT brand**	**Glaucoma**	**No. of participants**		**Gender (Male)**		**Age (Y), mean** **±** **SD**		**CDR, mean** **±** **SD**		**IOP (mm Hg)**		**Quality scaling (NOS)**
					**Case**	**Control**	**Case**	**Control**	**Case**	**Control**	**Case**	**Control**	**Case**	**Control**	
Lever et al. (34)	Germany	3	Heidelberg spectralis	PCG, JOAG, other types	19	53	NA	NA	11.2 ± 3.5	12.2 ± 3.5	0.8	0.6	18.7 ± 7.2	14.5 ± 2.7	7
Michelle et al. (31)	USA	1	Heidelberg spectralis	G/SG	39	57	22	28	5.9 ± 5.9	2.3 ± 1.5	NA	NA	23 ± 8	14 ± 3	7
Perucho-González et al. (32)	USA	2	Heidelberg spectralis	PCG	59	87	NA	NA	9.61 ± 3.23	8.47 ± 2.99	NA	NA	19.11 ± 4.23	13.95 ± 1.98	6
Pilat et al. (33)	UK	2	Envisu 2,300	PCG	20	20	10	11	4.64 ± 2.79	4.73 ± 2.81	0.668 ± 0.173	0.398 ± 0.178	17.68 ± 6.52	NA	8
Xu et al. (30)	USA	3	Heidelberg spectralis	PCG	20	15	11	6	9.7 ± 3.3	11.2 ± 3.3	0.4 ± 0.2	0.7 ± 0.1	15.4 ± 3.6	16.5 ± 3.6	8
Morales-Fernandez et al. (27)	Spain	2	Heidelberg spectralis	PCG	40	60	24	24	11.20 ± 3.94	10.90 ± 2.46	0.52 ± 0.29	0.24 ± 0.14	NA	NA	7
Silverstein et al. (28)	USA	NA	Heidelberg spectralis	PCG/JOAG	37	43	20	22	9.9 ± 3.3 (PCG) 13.1 ± 2.0 (JOAG)	11.1 ± 3.1 (PC) 12.3 ± 3.2 (N)	0.52 ± 0.29 (PCG) 0.83 ± 0.2 (JOAG)	0.7 ± 0.11 (PCG) 0.21 ± 0.12	NA	NA	6
Ghasia et al. (26)	USA	1	Optovue RTVue	G/SG	12	13	6	6	11.5 ± 3.5	10 ± 2.5	0.4 ± 0.2	0.1 ± 0.1	NA	NA	8
Srinivasan et al. (29)	India	4	Heidelberg spectralis	PCG	37	41	20	22	10.1 ± 3.6	13.6 ± 3.2	NA	NA	30.2 ± 5.9	NA	8
Ghasia et al. (25)	USA	1	Heidelberg spectralis	MG/M-to-SG	28	55	15	30	12 ± 1.08 (MG) 13 ± 1.6 (M-to-SG)	11 ± 0.75 (PC)12 ± 3 (N)	0.43 ± 0.04 (MG) 0.72 ± 0.04 (M-to-SG)	0.67 ± 0.02 (PC)0.15 ± 0.03 (N)	NA	NA	7

*1, Prospective cross-sectional study; 2, Observational cross-sectional study; 3, Retrospective observational case series; 4, Case–control study. OCT, optical coherence tomography; G, glaucoma; SG, suspect glaucoma; PCG, primary congenital glaucoma; JOAG, juvenile open-angle glaucoma; MG, mild glaucoma; M-to-SG, moderate-to-severe glaucoma; PC, physiologic cupping; N, normal; NA, not available; CDR, cup-to-disc ratio; IOP, intraocular pressure*.

### Meta-Analysis of Childhood Glaucoma Group Compared With a Healthy Control Group

**Figure 2:**
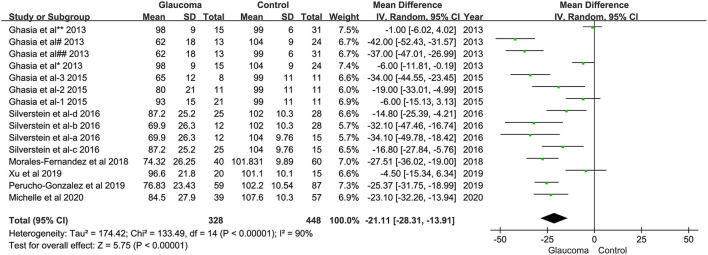
Forest plots of weighted mean difference (WMD) in the glaucoma group and healthy control group for average peripapillary retinal nerve fiber layer (RNFL) thickness. Horizontal lines are 95% CIs.

Analysis of average pRNFL thickness between the group of children with glaucoma and children without glaucoma in eight studies showed significant heterogeneity (I2 = 89%). Hence, the random effects model was used for data analysis. The meta-analysis showed that the average pRNFL thickness in the pediatric glaucoma group was decreased significantly than the non-glaucomatous group (WMD = −20.75; 95% CI −27.49–−14.01; *p* < 0.00001, Figure 2).

**Figure 3:**
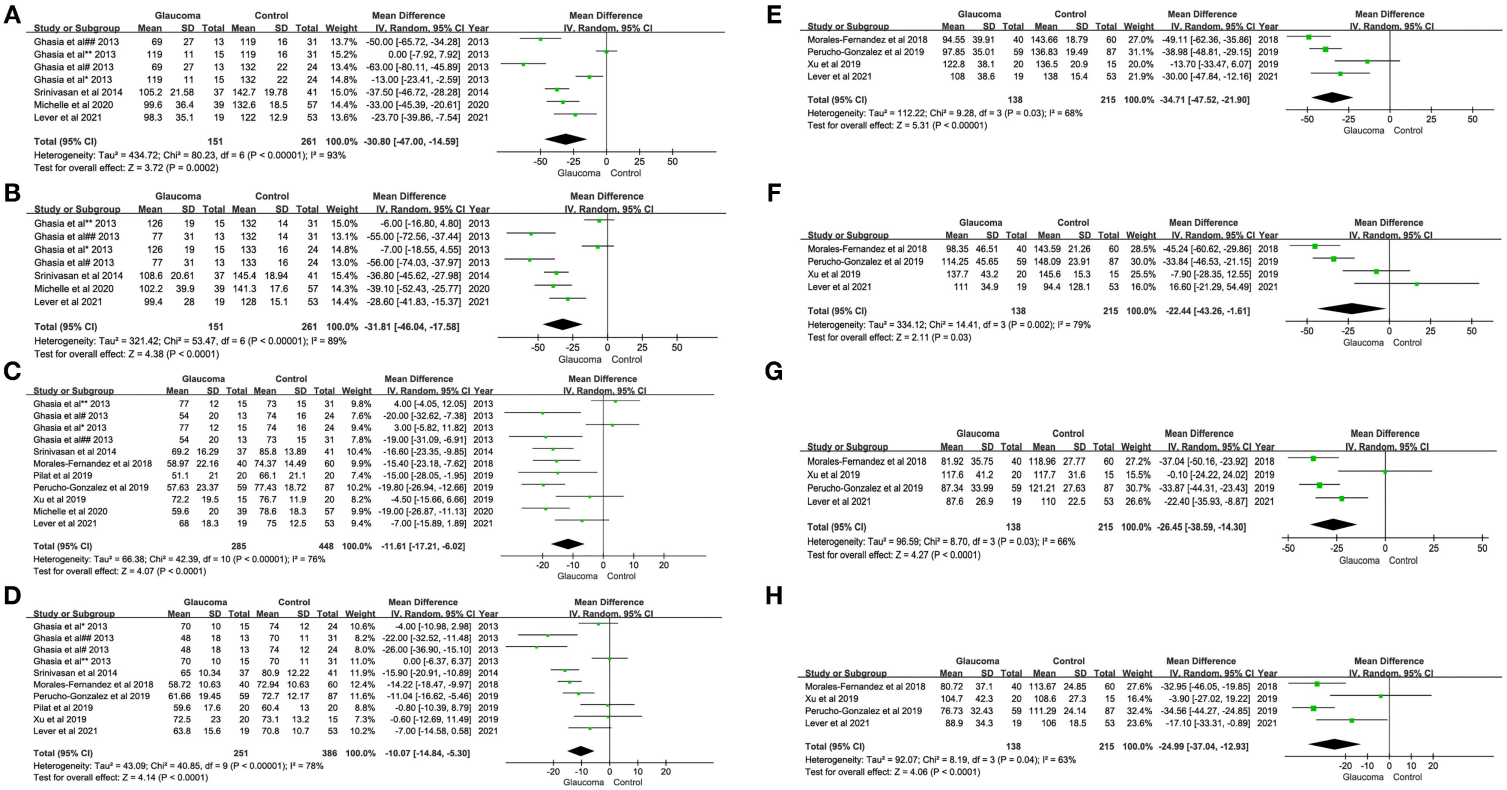
Forest plots of weighted mean difference (WMD) in the glaucoma group and healthy control group for peripapillary RNFL thickness in eight quadrants. Horizontal lines are 95% CIs. **(A)** Superior; **(B)** Inferior; **(C)** Nasal; **(D)** Temporal; **(E)** Superotemporal; **(F)** Inferotemporal; **(G)** Superonasal; and **(H)** Inferonasal. #, Moderate-to-severe glaucoma vs. normal; ##, Moderate-to-severe glaucoma vs. physiologic cupping; *, Mild glaucoma vs. normal; **, Mild glaucoma vs. physiologic cupping; 1, glaucoma suspect/preperimetric glaucoma vs. normal; 2, mild glaucoma vs. normal; 3, moderate/severe glaucoma vs. normal; a, JOAG vs. normal; b, JOAG vs. physiologic cupping; c, PCG vs. normal; d, PCG vs. physiologic cupping. RNFL, retinal nerve fiber layer; JOAG, juvenile open-angle glaucoma; PCG, primary congenital glaucoma.

In addition, we also examined the difference in pRNFL thickness between patients with glaucoma and healthy controls in each quadrant and there was significant difference in pRNFL thickness between those two groups in the superior (WMD = −30.80; 95% CI −47.00 to −14.59; *p* < 0.00001), inferior (WMD = −31.81; 95% CI −46.04 to −17.58; *p* < 0.00001), nasal (WMD = −11.61; 95% CI −17.21 to −6.02; *p* < 0.00001), temporal (WMD = −10.07; 95% CI −14.84 to −5.30; *p* < 0.00001), superotemporal (WMD = −34.71; 95% CI −47.52 to −21.90; *p* = 0.03), inferotemporal (WMD = −22.44; 95% CI −43.26 to −1.61; *p* = 0.002), inferonasal (WMD = −26.45; 95% CI −38.59 to −14.30; *p* = 0.03), and superonasal quadrant (WMD = −24.99; 95% CI −37.04 to −12.93; *p* = 0.04) between the glaucoma group and healthy control (Figure 3).

### Publication Bias

Due to the few eligible studies in our meta-analysis, funnel plots were easily seen as asymmetrical and we could not claim or deny bias for certain. Therefore, the funnel plot and Egger's statistics were not interpretable (data are not shown).

## Discussion

To the best of our knowledge, this review and meta-analysis are the first systematic synthesizing currently available observational studies to investigate the pRNFL thickness in childhood glaucoma. In this comprehensive meta-analysis, we investigated the specific regions of reduced pRNFL thickness in childhood glaucoma. As expected, the average pRNFL thickness was significantly thinner in the pediatric patients with glaucoma than in the healthy controls. There were also significant differences in pRNFL thickness between the groups in the superior, inferior, nasal, temporal, superotemporal, inferotemporal, superonasal, and inferonasal quadrants. We additionally observed the tendency of pRNFL thinning among the different regions in pediatric glaucoma of which the nasal and temporal quadrants demonstrated a small reduction in pRNFL thickness. The present meta-analysis showed that childhood glaucoma is strongly associated with changes in pRNFL thickness in the peripapillary region.

In our view, one of the important sources of high heterogeneity may have been the different inclusion criteria and adjusted factors across the studies. First, there were differences in types of childhood glaucoma and only the two pieces of literature mentioned the severity of glaucoma. Second, the exclusion criteria of individual studies differed greatly. For example, one study excluded patients with juvenile open-angle glaucoma (JOAG) that was considered an inherently progressive disease, while others included them or did not state whether JOAG was included. Additionally, the age range of childhood glaucoma was large, which may be a confounding variable in heterogeneity analysis. Third, although the pooled results of our study showed a significant decrease in superior, inferior, and superotemporal quadrants, we could not speculate which regions had the most significant reduction in children with glaucoma due to a large 95% CI, which is probably related to the severity of glaucoma.

It is reported that the pRNFL thickness in the superior and inferior quadrants is thicker and the temporal and nasal quadrants are relatively thinner in healthy adults (37–39). Turk et al. (40) evaluated 107 healthy Turkish children aged 6–16 years using SD-OCT and found that the average pRNFL thickness was 106.45 ± 9.41 μm, inferotemporal pRNFL (the thickest sector) thickness was 144.64 ± 17.16 μm, and nasal pRNFL (the thinnest sector) thickness was 71.54 ± 10.03 μm. Zhu et al. (41) examined 2,105 12-year-old healthy students using SD-OCT and reported that the average pRNFL thickness was 103.08 ± 1.2 μm and the pRNFL thickness was thicker with shorter axial length and higher hyperopia. Rotruck et al. (42) reported that the mean global pRNFL thickness of healthy children aged 0–5 years old was 107.6 ± 10.3 μm and it was not dependent on age. In general, the thickest region of pRNFL in healthy children is the inferior quadrant, which is thicker than in adults and not dependent on age but showed a negative relationship with axial length (41–44). The pRNFL thickness may attenuate subsequently because of physiologic growth in axial length during the growth and development of children. However, this corresponding change of normal eyes in pRNFL thickness is considerably less than the variability compared with pathological progression and should not be clinically significant (30, 45). Moreover, children with large CDRs were considered as the potential hazard of a glaucomatous process. However, the study (46) revealed that the degree of optic nerve cupping does not correlate with the RNFL thickness. Normal eyes and physiologic cupping showed equivalence in the several measurement parameters between all macular layers and pRNFL (28). Hence, the data can be analyzed by incorporating both normal controls and physiologic cuppingsimultaneously.

More attention should be given when the pRNFL thickness thinning exceeds approximately 8 μm because it is a likely sign of the probable clinical change (30). Compared to the other optic disc parameters, pRNFL thickness has a higher diagnostic accuracy of glaucoma, especially in the superior and inferior regions (27, 47). Studies have shown that RNFL thinning commonly begins with the inferior and superior sectors and then affects nasal and temporal regions in adult patients and the temporal RNFL quadrant is most conserved in glaucoma (48, 49). This phenomenon may be explained by the preservation of central vision in patients with glaucoma, which is mainly fed by the temporal RNFL fibers (50–52). The changes of pRNFL thickness are meaningful in these children with less severe glaucomatous optic nerve damage, where the damage of visual function may be difficult to measure. Thus, the superior and inferior pRNFL might be optimal to reflect glaucomatous changes, which is consistent with the results of our meta-analysis. In the adult patients with pseudoexfoliation glaucoma, one of the subtypes of open-angle glaucoma, the temporal RNFL thickness is not significantly different from the healthy controls. Our results, however, showed otherwise. In our meta-analysis, the temporal pRNFL thickness was thinning but the superonasal sectors were not different from normal eyes (48). This indicates that childhood glaucoma has its characteristics of RNFL thinning. Furthermore, a larger lamina cribrosa curvature index (LCCI) was perceived as the first indication of prognosis for faster progressive RNFL thinning. Primary congenital glaucoma (PCG) had significantly deeper cups than adults with primary open angle glaucoma emphasizing the importance of measuring cup depth besides concerning CDR (53, 54). Therefore, additional research is needed to demonstrate these differences with the development and preservation of visual function in childhood glaucoma and glaucoma in adults. Our focus should be especially on cup depth and morphology of lamina cribrosa testing RNFL thinning by SD-OCT in pediatric patients. Some of the included studies elucidated the stage of glaucoma (25, 26) and others were not (27–33), which may influence our meta-analysis of RNFL thickness. These could explain the high degree of statistical heterogeneity between the studies in the meta-analysis.

Spectral domain optical coherence tomography is a valuable tool in diagnosing and monitoring visual loss, especially for pediatric glaucoma. It provides an objective and quantitative scan with three-dimensional images, increased speed of image acquisition, and improved axial resolution for early diagnosis and monitoring of disease progression. This device has proved to be useful in the management of pediatric glaucoma as it provides objective RNFL thickness measurements in children (45). The advantages of SD-OCT compared to OCT devices in the past are short exposure durations and automatic eye-tracking systems. SD-OCT has higher sensitivity and specificity compared to the past OCT devices in detecting RNFL thickness. Its overall diagnostic precision of detecting and measuring localized RNFL defects in patients with glaucoma was also relatively higher than conventional fundus photographs (55, 56). Consequently, SD-OCT has potential clinical value in evaluating progression and follow-up for pediatric glaucoma as an effective means.

However, it was challenging to obtain high-quality images for children with glaucoma in the past due to poor cooperation, the opacity of refractive media, and unsuitable examination equipment. Poor-quality images often make it difficult for ophthalmologists to judge the progression of the disease. Delaying in diagnosis and treatment can increase the risk of irreversible optic nerve damage and even blindness. With the technical improvement and development of new instruments, the emergence of handheld OCT and overhead-mounted OCT solved this problem perfectly. The high-quality image can be acquired and analyzed by handheld and overhead-mounted OCT in very young patients. Updated SD-OCT has presented benefits for improved clinical management and study of the pathophysiology associated with childhood glaucoma (31, 33).

To some extent, there were several limitations to this study that need to be considered. First, the number of included studies as well as the included pediatric patients with glaucoma in each study was relatively small. In addition, childhood glaucoma is rare and there are some limitations to acquire good-quality imaging such as nystagmus or poor media opacity. Second, the severity of glaucoma was verified to be related to the mean pRNFL global thickness in children older than 6 years (40, 44), but we were unable to analyze the association between pRNFL thickness and the severity of pediatric glaucoma due to lack of sufficient studies providing data on the severity of glaucoma. Third, included studies of this meta-analysis were applied in four different SD-OCT devices (8 Heidelberg, 1 Envisu, and 1 Optovue). A primary concern was the measurement differences between manufacturers, which were statistically significant in the past and the clinical implication of this difference is less clear (57). However, this study believed that there were no statistically significant differences between devices for any particular area imaged and no major differences were noted for any of the parameters across OCT devices (58). Fourth, as a non-negligible factor, axial length affects RNFL measurement in the direction of thinner RNFL because of physiological growth or pathological factors such as high myopia (59). Therefore, future studies should obtain a random distribution of myopic and non-myopic participants in both normal and glaucomatous subgroups. Fifth, although, we had demonstrated specific regions of thinning pRNFL in pediatric glaucoma, the lack of studies about early-stage changes and progression in childhood glaucoma is a current limitation.

## Conclusion

In summary, our meta-analysis indicates that patients with childhood glaucoma have significantly reduced average eight quadrants RNFL thickness compared with healthy controls. We also observed a non-uniformity in the thinning of the pRNFL in different quadrants. Therefore, we suggest that the segmentation of peripapillary RNFL thickness as measured by SD-OCT can provide valuable information for the diagnosis and follow-up of childhood glaucoma. Moreover, a standardized and reproducible measurement technique of RNFL thickness needs to be introduced and utilized in children. In future research, further studies with multicenter and larger sample sizes, prospective and longitudinal studies are required to confirm the present results of this meta-analysis.

## Data Availability Statement

The original contributions presented in the study are included in the article/supplementary material, further inquiries can be directed to the corresponding author/s.

## Author Contributions

YW was responsible for designing the review protocol, writing the protocol and report, conducting the search, screening potentially eligible studies, extracting and analyzing data, interpreting results, updating reference lists, and creating figures and tables. YiZ was responsible for designing the review protocol and screening potentially eligible studies and contributed to writing the report, extracting and analyzing data, and interpreting results. YeZ contributed to the design of the review protocol, arbitrating potentially eligible studies, extracting and analyzing data, and interpreting results provided feedback on the report. All authors listed have made a substantial, direct and intellectual contribution to the work, and approved it for publication.

## Funding

This study was supported by the National Key Research and Development Project of China (grant number 2020YFA0112701) and the National Natural Science Foundation of China (81700858 and 81870658).

## Conflict of Interest

The authors declare that the research was conducted in the absence of any commercial or financial relationships that could be construed aspotential conflicts of interest.

## Publisher's Note

All claims expressed in this article are solely those of the authors and do not necessarily represent those of their affiliated organizations, or those of the publisher, the editors and the reviewers. Any product that may be evaluated in this article, or claim that may be made by its manufacturer, is not guaranteed or endorsed by the publisher.
